# Mitochondrial heterogeneity and crosstalk in aging: Time for a paradigm shift?

**DOI:** 10.1111/acel.14296

**Published:** 2024-08-26

**Authors:** Antentor O. Hinton, Zer Vue, Estevão Scudese, Kit Neikirk, Annet Kirabo, Monty Montano

**Affiliations:** ^1^ Department of Molecular Physiology and Biophysics Vanderbilt University Nashville Tennessee USA; ^2^ Department of Medicine Vanderbilt University Medical Center Nashville Tennessee USA; ^3^ Vanderbilt Center for Immunobiology Nashville Tennessee USA; ^4^ Immunology and Inflammation Vanderbilt Institute for Infection Nashville Tennessee USA; ^5^ Vanderbilt Institute for Global Health Nashville Tennessee USA; ^6^ Department of Medicine Harvard Medical School Boston Massachusetts USA

**Keywords:** aging, mitochondria, organelle contacts, protein targeting, structure

## Abstract

The hallmarks of aging have been influential in guiding the biology of aging research, with more recent and growing recognition of the interdependence of these hallmarks on age‐related health outcomes. However, a current challenge is personalizing aging trajectories to promote healthy aging, given the diversity of genotypes and lived experience. We suggest that incorporating heterogeneity—including intrinsic (e.g., genetic and structural) and extrinsic (e.g., environmental and exposome) factors and their interdependence of hallmarks—may move the dial. This editorial perspective will focus on one hallmark, namely mitochondrial dysfunction, to exemplify how consideration of heterogeneity and interdependence or crosstalk may reveal new perspectives and opportunities for personalizing aging research. To this end, we highlight heterogeneity within mitochondria as a model.

## Graphical abstract

This editorial highlights intrinsic and extrinsic factors that may contribute to mitochondrial heterogeneity and quality control mechanisms. As depicted in the graphical abstract, these factors may affect mitochondrial metabolism, mitochondrial structure, mitochondrial quality control, crosstalk with other organelles, mitochondrial DNA quality, and epigenetic regulation, pluralistically affecting the hallmarks of aging.

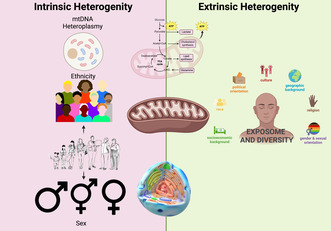

**Abbreviations**
ER: endoplasmic reticulum
MERC: mitochondria–endoplasmic reticulum contact site
mtDNA: mitochondrial DNA

## 1 INTRODUCTION

Understanding and operationalizing the hallmarks of aging present significant challenges, when considering the diversity of life histories and environmental settings that influence age trajectories of people and populations. The molecular drivers of biological aging, initially presented as a discrete set in 2013 by López‐Otín continue to expand (López‐Otín et al., 2023). However, they do not directly address the role of crosstalk between hallmarks and the potential contributions biological heterogeneity has on age‐related health outcomes. As suggested by Dan Ehninger and colleagues (Keshavarz et al., 2023), evaluating the biology of aging solely based on lifespan metrics undervalues the effect of healthspan‐related pathologies (Keshavarz et al., 2023). This idea aligns with recent findings that organs do not necessarily age at the same rate (Klug et al., 2021; Moqri & Snyder, 2023; Vougioukalaki et al., 2022) and, by extension, organ‐associated diseases of aging. In one study, 20% of an adult cohort showed accelerated single‐organ aging, conferring increased mortality risk (e.g., accelerated heart aging with an increased risk for Alzheimer's disease and a 2.5‐fold likelihood of developing heart failure) (Oh et al., 2023), underscoring a potential disconnect between single organ age and integrative whole organism outcomes. In a recent review by Skowronska‐Krawczyk, integrative models for the hallmarks of aging are necessary to compare interindividual differences (Skowronska‐Krawczyk, 2023). In their model, rather than independent markers, hallmarks should be conceptualized as interdependent “three‐wheeled gears,” such that, early molecular changes arise from both inter‐ and intra‐individual differences or environmental cues, causing broader cellular and tissue responses (Skowronska‐Krawczyk, 2023).

With the growing recognition that aging is a highly heterogeneous process influenced by genetic, environmental, and lifestyle factors, strategies for operationalizing the hallmarks of aging will need to account for this heterogeneity if they are to be useful in clinical and public health practice. Thus, aging research has begun to shift towards a life‐course perspective that considers early‐life exposures and their long‐term impact on aging trajectories and hallmark dysfunction (e.g., mitochondrial dysfunction) (Bazopoulou et al., 2019). This editorial focuses on age‐dependent mitochondrial changes within the context of intrinsic heterogeneity (e.g., genetic and structural variation) and extrinsic heterogeneity (i.e., from inter‐organelle to exposome to social determinants of health), underscoring the value of recognizing mitochondrial crosstalk with other aging hallmarks and organelles.

**FIGURE 1:**
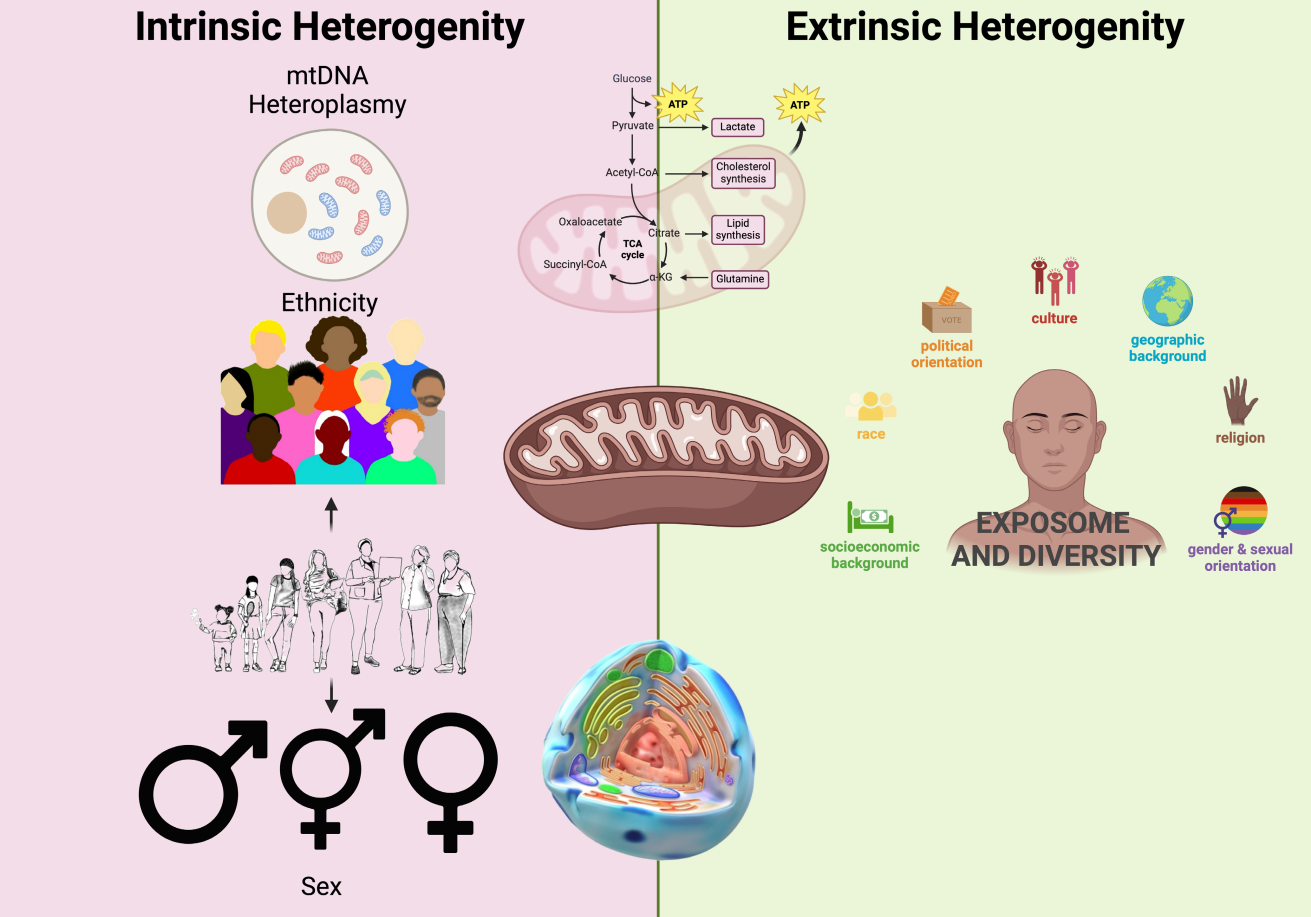
Examples of intrinsic and extrinsic factors that may contribute to mitochondrial heterogeneity and quality control mechanisms. These factors may affect mitochondrial metabolism, mitochondrial structure, mitochondrial quality control, crosstalk with other organelles, mitochondrial DNA (mtDNA) quality, and epigenetic regulation, pluralistically affecting the hallmarks of aging.

As previously reviewed (Aryaman et al., 2018; Chen et al., 2023; Naik et al., 2019), mitochondrial heterogeneity encompasses variations in morphology; that is, composition and expression of proteins and enzymes; and activities such as ATP production, redox state, and calcium handling. These features, along with genetic mutations, epigenetic modifications, altered metabolic states, cellular stress responses, and exposure to environmental stressors have tangible impacts on physiological processes and potential therapeutic strategies for various pathological conditions (Chen et al., 2023). Notably, genotypical changes arising from ancestral differences in maternally‐inherited mitochondrial DNA (mtDNA) can lead to intrinsic heterogeneity (Kivisild, 2015). Additionally, interindividual genotypic variation may be compounded by wider contextual variation with the exposome, which consists of the environment, social factors, and other external regulators that contribute to heterogeneity in mitochondria function, and by extension, health‐related outcomes (Figure 1).

### 1.1 Intrinsic heterogeneity and mitochondrial function

Increasingly, metabolic responses to diet‐mediated lifespan extensions have been linked to genotype (Jin et al., 2020) and sex (Green et al., 2022), showing these intrinsic factors may serve principle roles in interindividual heterogeneity. Elucidating intrinsic heterogeneity of mitochondria across the life course, with consideration to biological sex and genotype (i.e., ethnicity), is therefore important for better understanding the role of mitochondria in age‐related outcomes.

A review by Hägg & Jylhävä on sex differences in biological aging highlighted the complex nature of biological aging mechanisms influenced by sex, with an emphasis on distinguishing lifespan from healthspan (Hägg & Jylhävä, 2021). Mitochondrial dysfunction may contribute to sex and age‐dependent differences, as exemplified by differences in mitochondrial functions, including calcium uptake, between men and women (Demarest & McCarthy, 2015). However, a broad meta‐analysis by Junker and colleagues of sex‐dependent differences points toward a small‐to‐moderate, age‐independent effect relegated to increased mitochondrial content and decreased oxidative stress in specific tissue types (Junker et al., 2022). And still, other studies provide more convincing evidence of a sex‐dependent difference, underscoring the need for longitudinal studies incorporating sex and gender, as well as age. Studies using skeletal muscle fibers indicate that intermyofibrillar mitochondrial size is reduced across aging predicated on a sex interaction effect (Callahan et al., 2014), which suggests a clear interaction between mitochondrial structure and sex‐dependent, age‐related changes. Similar results have been recapitulated in cardiac tissue, showing that sex hormones regulate mitochondrial function and mtDNA levels, directly impacting sex‐dependent differences in diastolic function (Cao et al., 2022). In the brain, not only do metabolic indicators differ between men and women, but so too does mitochondrial content with intraindividual regional content differences in white matter compared to gray matter (Gaignard et al., 2015; Silaidos et al., 2018). A recent study found that sex‐specific differences in mitochondrial function, morphology, and redox status are observed early in life, potentially implicating sexual dimorphism in cardiac and neurological disorders, with females showing enhanced respiration and lower reactive oxygen species production compared to males (Khalifa et al., 2017). Collectively, these studies indicate that mitochondrial sex dependency may be specific to organs or organ regions and that biological heterogeneity (both organ and whole body) needs to be incorporated into future studies of age and sex effects on healthspan.

In addition to interindividual heterogeneity, the location of mitochondria within the cell may confer intraindividual differences in activity, redox state, and calcium uptake (Kuznetsov & Margreiter, 2009). For example, in the context of skeletal muscle, perinuclear, subsarcolemmal and, intermyofibrillar mitochondria skeletal muscle display differential oxidized states, membrane potential, arrangement, and morphology (Kuznetsov et al., 2006). Willingham et al. (2021), along with studies by Glancy et al. (2020) and Jenkins et al. (2024), comprehensively examine variations in mitochondrial shape and their functional implications, underscoring intra‐individual heterogeneity (Glancy et al., 2020; Jenkins et al., 2024; Willingham et al., 2021). Tissue‐dependent differences suggest that relative mitochondrial location and subpopulations may differ due to sex‐dependent differences across the life course, but while some studies confirm this, the overall influence of sex, both between and within individuals, especially in subcellular mitochondria specialization, will certainly require more research.

Another field of intrinsic heterogeneity that remains understudied but is gaining traction is the link between mitochondrial heterogeneity and genotypic variation (e.g., ethnicity or genetic background). A probable link is through genetic polymorphisms, such as single‐nucleotide polymorphism, which are understood to vary across ethnic populations and confer differential risk to some disease states (Pande et al., 2014). Polymorphisms in genes such as SLC44A1 offer a genetic foundation for understanding mitochondrial function, preceding their modulatory effects (da Costa et al., 2014). In European Americans, mitochondrial single‐nucleotide polymorphisms are associated with an increased risk of colorectal cancer (Li et al., 2015). These findings support the abundance of studies that have used repositories of biological samples for research (i.e., biobanks) to show that ethnicity may influence specific biological pathways and functions. AutoMitoC has been used as a novel tool for genome‐ and exome‐wide association studies across nearly 400,000 UKBiobank to show that mtDNA copy number, differed between ethnicities, with South Asians having significantly lower levels than Europeans, and intrinsic factors (e.g., age, sex, and ethnicity) accounting for 0.83% of variance in mtDNA copy number (Chong et al., 2022). While mtDNA mutations, which may be deleterious and contribute to the dysregulation of mitochondrial quality control mechanisms, can be maternally inherited, they can also accumulate with aging (Liu et al., 2024). This age‐related damage confers another source of intrinsic heterogeneity, while also potentially linking genetic heterogeneity to late‐onset diseases (Stewart & Chinnery, 2015). Studies have also used whole mtDNA sequencing to elucidate ethnicity‐dependent differences due to an apparent mtDNA bottleneck during human migration, resulting in the vast majority of non‐African mtDNA lineages deriving from the African haplogroup L3 (Kivisild, 2015). While outside the scope of this editorial, an additional complication and source of potential biological variation in mitochondrial function is the role of epigenetic factors that regulate mtDNA replication, heterogeneity, and quality that need to be considered across genotypical backgrounds (Sharma et al., 2019).

Complicating the study of mtDNA inter‐individual differences is a tremendous intra‐individual variability in mtDNA. Mitochondrial diseases may occur due to extensive heteroplasmy within and between individual mitochondria (Morris et al., 2017); although not universally observed (Sanchez‐Contreras et al., 2023; Vandiver et al., 2023). This presents an apparent paradox in the field of mtDNA research characterized by a discrepancy between observed frequencies in mtDNA genetic variation and functional consequences. While data from murine (Sanchez‐Contreras et al., 2023) and human (Vandiver et al., 2023) aging studies describe age‐related mitochondrial mutation/deletion frequencies of approximately 0.0001% and <0.01%, respectively, functional assessments often reveal near‐complete deletion and mutation frequencies (Kandul et al., 2016). Additionally, despite mtDNA heteroplasmy being remarkably common (Stewart & Chinnery, 2015), investigations into inherited mitochondrial disorders suggest a threshold effect, wherein a significant proportion of mtDNA genomes must be affected before observable phenotypic expression occurs (Miyabayashi et al., 1992). This seeming paradox may be reconciled by considering selective and regulatory pressures, as previously discussed (Latorre‐Pellicer et al., 2019), with mito‐nuclear interactions regulating these changes. Additionally, it is possible that cells with high levels of damage may be eliminated through mitochondrial quality control mechanisms (e.g., mitophagy), thereby preventing their accumulation to higher levels.

Thus, our understanding of the extent and functional outcomes of intrinsic heterogeneity while growing still remains limited. Though pathologies can affect the mitochondrial structure (Chan, 2006; Chen et al., 2023; Duchen & Szabadkai, 2010), it remains controversial whether and how certain demographic factors attenuate or potentiate age‐related structural or functional alterations. The implications for personalized or stratified medicine approaches in biogerontology based on mitochondrial heterogeneity will likely rely on developing technologies and scoring systems for characterizing and incorporating heterogeneity into functional studies, from organ‐specific variation to integrative biology of whole persons and populations.

### 1.2 Extrinsic heterogeneity and mitochondrial function

In addition to intrinsic heterogeneity, there is a growing interest in the role for environmental variation on mitochondrial function, collectively viewed as the exposome (i.e., broad environmental exposures that can include various exposures such as diet (Putti et al., 2015) toxins (Meyer et al., 2013), as well as less tangible factors such as experienced psychosocial or biological stress). Because health disparities in age‐related diseases are observed in association with demographics including race and socioeconomic statuses (SES) (Noren Hooten et al., 2022), the potential interplay between lifestyle exposures and mitochondrially associated age‐related outcomes will also need to be considered and is a research opportunity.

Notably, although race is a social construct, the biological effects that can occur from a lifetime of systematic racism likely influence risk for adverse health outcomes. Societal stressors including John Henryism (Rolle et al., 2021) [i.e., exposures where the allostatic load or balance of stressors to reserves is unfavorable (Guidi et al., 2020)] would benefit from specific mechanistic linkage to health outcomes. Mitochondrial allostatic load can be viewed as an alteration in the rates of energy utilization with aging (Picard & McEwen, 2018), and raises the possibility that social determinants may directly influence mitochondrial function, by as yet unclear mechanistic pathways. As one example, epidemiologic studies of breast cancer patients indicate that non‐White individuals, divorced individuals, non‐college‐educated individuals, smokers, and individuals with low levels of physical activity, have a higher allostatic load that can be linked to variation in the number of mtDNA copies per cell (Zhao et al., 2021). This highlights a potential interdependence where social determinants of health and lifestyle behaviors may be linked to increases in mtDNA variation. Relatedly, self‐reported health status correlates with mtDNA copy number (Takahashi et al., 2018). In a large study of 13,189 heteroplasmies comparing maternal lifetime stress with mitochondrial mutational load, Black women were reported to be particularly vulnerable to increased psychosocial stress and increased mitochondrial mutational load (Brunst et al., 2021). Recent studies suggest that mtDNA deletions and mtDNA copy number variation accumulate with aging and can modify the risk for psychiatric and neurological diseases (Valiente‐Pallejà et al., 2022). While one study suggested that no specific mtDNA alteration could be used as a disease marker, a study of almost 1000 mitochondrial single‐nucleotide polymorphisms showed that mt12501G→A and mt8414C→T heteroplasmy increases the risk of post‐traumatic stress disorder (Flaquer et al., 2015) linking health outcomes to mitochondrial variation. Not all stress exposures result in mtDNA heterogeneity. In a study of over 100 holocaust survivors and their descendants, no significant difference were observed in mtDNA copy number among any groups, regardless of, as compared to ethnically‐matched controls (Cai et al., 2020). Another important area for research is intraindividual variation in mutational burden across organs. One study found that brain samples displayed more drastic phenotypes and elevated levels of mtDNA deletions and heteroplasmy, when compared to blood samples, and with variations in mutational burden even observed across different brain regions (Valiente‐Pallejà et al., 2022). Finally, it remains unclear how transgenerational high allosteric loads of mtDNA may pass across generations (i.e., generational trauma). Non‐mtDNA mechanisms of intergenerational mitochondrial dysfunction remain similarly unclear and an opportunity for future studies. Indeed, one study has found in mice that intergenerational trauma, even when controlling for early‐life parenting, mitochondrial hypoxia marker, and epigenetic modifier 2‐hydroxyglutaric were increased, and that depression‐risk induced by these changes could be impeded by early intervention of acetyl‐L‐carnitine (Alhassen et al., 2021). These findings underscore the necessity for a comprehensive investigation into the roles of mitochondria in the acceleration of aging associated with experienced stress and inherited intergenerational stress, particularly in light of potential synergistic effects with other exposome factors (e.g., diet, toxin exposures, and physical activity levels) (Breton et al., 2019; Hajat et al., 2015; Oude Groeniger et al., 2019).

An additional consideration in studies of mitochondrial heterogeneity is the evaluation of the inter‐connectome [i.e., organelle contacts and interactions, as previously defined by Lee et al. (2023)]. Studies of the inter‐connectome could investigate tissue‐dependent changes in protein composition and their interacting players, modifications, and locations within cells and tissues—in the context of heterogeneity, both intraindividual (i.e., tissue‐dependent changes) and interindividual (e.g., in individuals with differential environmental or exposome exposures) differences. Specifically, investigation of protein expression across allostatic load variations could consider: (1) spatiotemporal structure and orientation using techniques such as mass spectrometry imaging (Hogan et al., 2023; Ong & Mann, 2005); (2) frequency of post‐translational modifications that may affect protein function and stability using techniques such as liquid chromatography–tandem mass spectrometry (Jensen, 2004); (3) relative expression of isoform‐specific proteins arising from alternative splicing, as measured by techniques such as isoform‐specific mass spectrometry (Tress et al., 2007); and (4) frequency and constitution of protein–protein interactions, as measured by proximity ligation assays (Hegazy et al., 2020).

### 1.3 Mitochondrial crosstalk

Mitochondrial function can be mechanistically linked to most if not all hallmarks of aging discussed by López‐Otín and colleagues (López‐Otín et al., 2023), and likely contributes to downstream events, as described by Skowronska‐Krawczyk (2023). For example, loss of mitochondrial phosphatase PGAM5 inhibits dephosphorylation of DRP1, a mitochondrial fission protein (Yu et al., 2020), thereby reducing mitochondrial turnover, activating mTOR signaling pathways, and ultimately triggering cellular senescence. Stem cell fate is also intrinsically tied to mitochondrial metabolism, redox signaling, and mitochondrial dynamics (Chakrabarty & Chandel, 2021). Furthermore, mitochondrial dysfunction precedes telomere attrition as evidenced by telomere loss and chromosome breakage following mitochondrial dysfunction (Liu et al., 2002). Central to many of these mitochondrial changes are oxidative stress and damage from mitochondria, which contribute to damage‐associated molecular patterns (i.e., DAMPs) and lead to activation of innate immune responses with associated inflammation (Jang et al., 2018). A key mechanism to counteract mitochondrial degeneration and mitigate the associated inflammation is through mitochondria‐specific autophagy (i.e., mitophagy), and notably, the loss of genes associated with mitophagy can increase vulnerability to age‐related diseases, such as Parkinson's disease (Green et al., 2011; Rambold & Lippincott‐Schwartz, 2011). Furthermore, alterations in epigenomic patterns of acetylation/methylation of mtDNA associated with aging, influenced by extrinsic factors, such as stress (Matilainen et al., 2017), may serve as an imprinting molecular memory, linking environmentally‐induced stressors with mitophagy. Despite mitochondrial dysfunction being recognized as a hallmark of aging, its intricate interplay with other hallmarks underscores the complexity of the interconnected regulatory mechanisms involved in mitochondrial function with aging. This perspective serves to highlight the potential value of including intrinsic/extrinsic heterogeneity in mitochondrial (and other hallmarks) in future studies.

**FIGURE 2:**
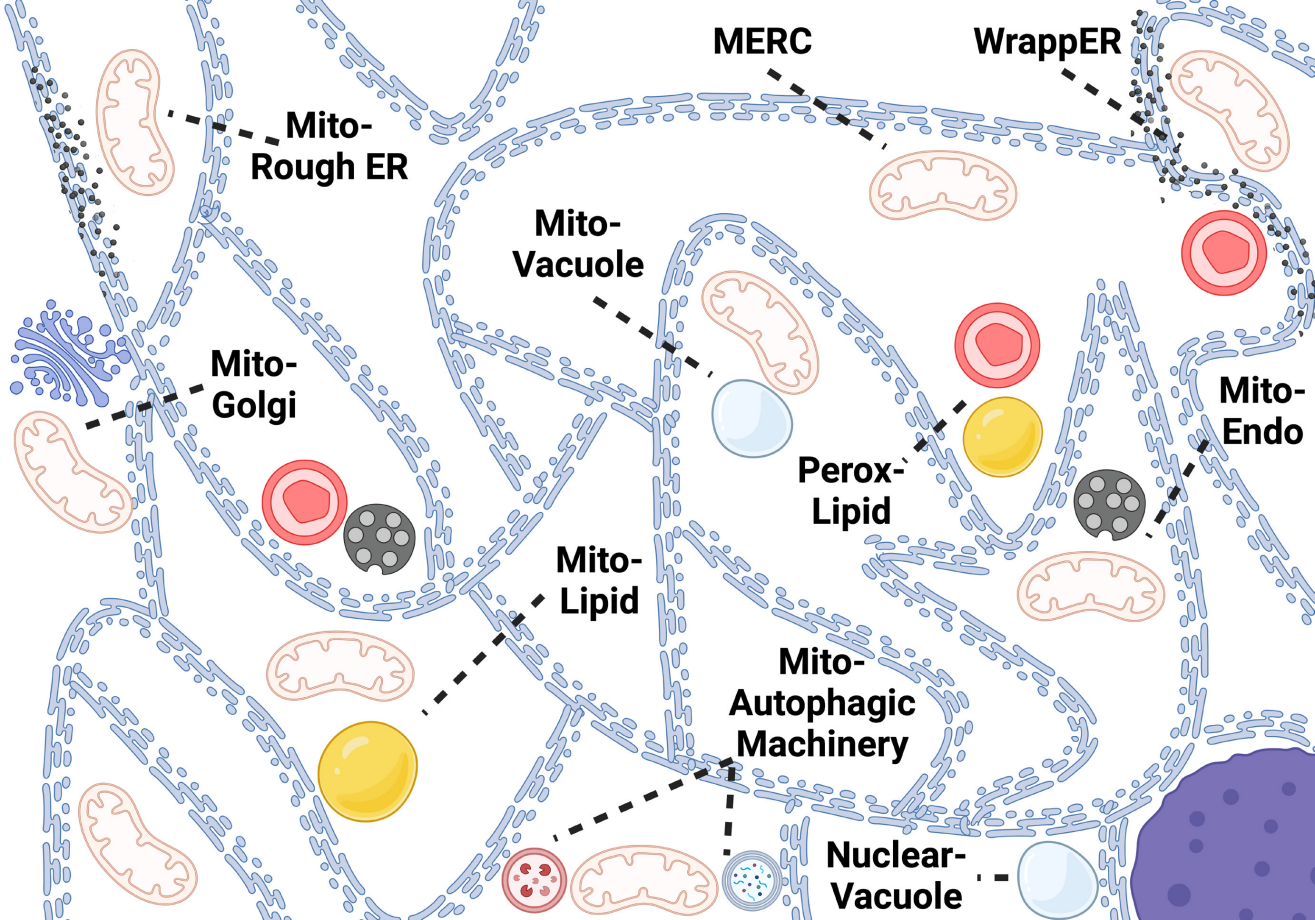
Endoplasmic reticulum (ER)‐connectome links mitochondria with contact sites, which may include two or more organelles. Spanning from the nucleus (bottom right), the ER (blue) engages with various organelles. Mitochondria endoplasmic reticulum contact sites (MERCs) regulate mitochondrial fission (e.g., SEL1L), lipid transfer (e.g., Mmm1, Mdm12, Mdm34), and calcium homeostasis (e.g., IP3R3, GRP75), while the space between these organelles constitutes the mitochondria‐associated membrane fraction (Silva et al., 2020). The MERCs' phenotypes may dictate their specific functions (e.g., whether it is formed with rough or smooth ER) or whether a 3‐organelle interaction will occur (e.g., WrappER). Some ER–organelle contacts, such as nucleus‐vacuole junctions, which are involved in lipid homeostasis and selective nuclear degradation (e.g., Nvj1, Vac8), do not involve the mitochondria. ER, endoplasmic reticulum; MERC, mitochondria–endoplasmic reticulum contact site; Mito, mitochondria; Golgi, golgi apparatus; Perox, peroxisome; Endo, endosome.

In addition to signaling pathway crosstalk between mitochondrial and other hallmarks, the subcellular spatial location of mitochondria may further influence age‐related changes, and crosstalk in mitochondria may be modulated by the organelle‐organelle interactions they form. Organelle interactions, including those formed by mitochondria in response to aging or cellular stress responses, may allow cells to respond to different environmental or developmental cues (Cohen et al., 2018). For example, mitochondria display fundamental structural changes during mitochondrial division (Tábara et al., 2021). Furthermore, mitochondria endoplasmic reticulum contact sites (MERCs) are multiple networked structures (Figure 2) that interact to carry out various homeostatic processes (Murley & Nunnari, 2016). We and others are actively studying how to measure these organelle–organelle contact changes per tissue in the context of biological aging (Hinton et al., 2023; Lam et al., 2021; Marshall et al., 2023; Neikirk et al., 2023). The endoplasmic reticulum (ER) engages in many organelle interactions, that include mitochondria in interactions under study (Figure 2) (Silva et al., 2020). For example, under conditions such as liver lipid flux, MERCs can transform into 3‐organelle contact sites accompanied by morphological changes in the ER (Ilacqua et al., 2021). MERCs can also vary in length, distance (i.e., morphology and ultrastructure), and metabolic activity, with loss of MERC‐associated proteins activating the unfolded protein response (Gordaliza‐Alaguero et al., 2019). An intriguing area of future work could involve deciphering whether and how shifts in MERC compositional details and biochemical activity may provide insight into age‐associated conditions and loss in homeostasis (Anastasia et al., 2021). As more organelle–organelle contact sites are uncovered, it will be important to explore new therapeutic avenues for remodeling the inter‐connectome in the context of tissue and organismal aging (Csordás et al., 2018; Oh et al., 2023). Similarly, future research focused on healthspan across diverse populations would benefit from including life history and allostatic load as potential drivers or modulators in the composition and distribution of MERCs and other organelles‐organelle contact states (Murley & Nunnari, 2016; Silva et al., 2020).

We have previously examined the tissue‐dependent changes in mitochondrial 3D ultrastructure and potential dynamic regulators associated with age‐related changes (Crabtree et al., 2023; Vue, Garza‐Lopez, et al., 2023; Vue, Neikirk, et al., 2023). These studies offer insight into not only the differences at baseline between mitochondrial structures in many different organs but also the pleiotropic changes they undergo in response to the aging process, thus providing potential insight into unique aging‐associated pathologies. However, along with other hallmarks, this approach has not yet been applied to many organelles and the wider organelle inter‐connectome. Other researchers have utilized techniques including correlative light electron microscopy with machine‐learning techniques to reconstruct the entire connectome and evaluate organelle‐interactions on a large scale (Cohen et al., 2018; Heinrich et al., 2021; Lee et al., 2023; Valm et al., 2017; Xu et al., 2021). Additionally, while methods of electron microscopy can be useful for high architectural detail and 3D reconstruction, super‐resolution live‐cell can allow for measuring millisecond‐scale changes in organelle interactions (Guo et al., 2018). Nascent techniques for superresolution analysis that may define organelle inter‐connectome contact sites include call expansion microscopy (Chen et al., 2015) and stochastic optical reconstruction microscopy (Huang et al., 2008). Additionally, rapid advances in techniques spanning from next‐generation sequencing approaches for mtDNA [reviewed in (Legati et al., 2021)] to in vitro spectrophotometric enzymatic assays to evaluate function [reviewed in (Marín‐García, 2013)], facilitate a study of mitochondria in greater detail than ever, allowing us to answer previously unanswerable questions.

### 1.4 Concluding comments

Although the updated hallmarks of aging (López‐Otín et al., 2023) include mitochondrial dysfunction, this editorial perspective highlights the need to also consider heterogeneity and interconnectedness of mitochondria with many (if not all) hallmarks of aging. The nuances of organelle dysfunction and organelle–organelle contacts in a context of life history and environmental exposures offer an opportunity to translate our understanding of how intrinsic and extrinsic factors shape mitochondrial dysfunction into actionable molecular profiles that could be useful in optimizing health trajectories. The emerging field of stress‐induced epigenetics is likely to inform intrinsic and extrinsic factor‐driven effects. Although research has increasingly shown that intrinsic factors, including sex and genotype, can affect mtDNA and mitochondrial function, studies on the intra and interindividual regulatory mechanisms, and how they translate to disease risk states, remain understudied yet vital to the mission of translational geroscience. Similarly, although extrinsic factors, including SES, may affect healthcare outcomes, it will be important to delineate which specific lifestyle and human exposome factors are relevant to geroscience and their mechanism(s) of action. Collectively, we posit that an interconnected view of mitochondria, considering how intrinsic and extrinsic factors coalesce to influence aging‐related processes and outcomes has significant untapped value in the application of translational geroscience.

## AUTHOR CONTRIBUTIONS

All authors contributed to the writing, revising, and final approval of the manuscript. AHJ and MM conceptualized and oversaw the project.

## CONFLICT OF INTEREST STATEMENT

None of the authors have conflicts of interest related to this article.

## Data Availability

None.
